# Early-onset breast cancer in a woman with a germline mobile element insertion resulting in *BRCA2* disruption: a case report

**DOI:** 10.1038/s41439-020-00111-z

**Published:** 2020-08-25

**Authors:** Natalie Deuitch, Shao-Tzu Li, Eliza Courtney, Tarryn Shaw, Rebecca Dent, Veronique Tan, Lauren Yackowski, Rebecca Torene, Windy Berkofsky-Fessler, Joanne Ngeow

**Affiliations:** 1 Cancer Genetics Service, Division of Medical Oncology, National Cancer Centre, Singapore, Singapore; 2 Department of Genetics, Stanford University School of Medicine, Stanford, CA USA; 3 Division of Medical Oncology, National Cancer Centre, Singapore, Singapore; 4 Division of Breast Surgical Oncology, National Cancer Centre, Singapore, Singapore; 5 Hereditary Cancer Program, GeneDx, Gaithersburg, MD USA; 6 Lee Kong Chian School of Medicine, Nanyang Technological University, Singapore, Singapore

**Keywords:** Cancer genomics, Next-generation sequencing

## Abstract

Mobile element insertions (MEIs) contribute to genomic diversity, but they can be responsible for human disease in some cases. Initial clinical testing (*BRCA1*, *BRCA2* and *PALB2*) in a 40-year-old female with unilateral breast cancer did not detect any pathogenic variants. Subsequent reanalysis for MEIs detected a novel likely pathogenic insertion of the retrotransposon element (RE) c.7894_7895insSVA in *BRCA2*. This case highlights the importance of bioinformatic pipeline optimization for the detection of MEIs in genes associated with hereditary cancer, as early detection can significantly impact clinical management.

Hereditary breast and ovarian cancer (HBOC) syndrome, caused by monoallelic germline pathogenic variants (PVs) in *BRCA1* (MIM# 604370) and *BRCA2* (MIM# 600185), is predominantly associated with an increased risk of breast and ovarian cancer in females^1^. Prompt detection of *BRCA1*/*2* PVs in women allows for surveillance and/or risk-reducing (RR) strategies, which have been demonstrated to reduce cancer-related morbidity and mortality^1^. Furthermore, knowledge of a woman’s *BRCA1*/*2* status following a diagnosis of cancer can inform surgical decision-making, such as pursuing RR contralateral mastectomy (RRCM) or RR bilateral salpingo-oophorectomy (RRBSO), and may guide therapeutic decisions, such as platinum-based neoadjuvant chemotherapy or poly ADP-ribose polymerase inhibitors^2,3^. Variant detection is also important for clarifying risk for family members^4^. While advances in sequencing technology and analysis have improved the detection of *BRCA1*/*2* PVs, testing is uninformative for a number of breast and ovarian cancer cases with high clinical suspicion^5,6^. Some of these cases may be explained by PVs in other cancer predisposition genes^7^. However, some of these individuals may carry PVs in the tested genes that were not detected by sequencing technologies at the time^4^.

Mobile element insertions (MEIs) are one of these classes of variants. They occur when transposable elements (TEs) “*jump*” to alternate locations in the genome through DNA transposases or reverse transcription^8–10^. There are many subsets of TEs, but in humans, long interspersed element-1s function as retrotransposons. They are copied into RNA and reinserted into the DNA by a reverse transcriptase enzyme^9^. They are capable of copying not only their own sequence but also other elements such as Alu elements, short interspersed element-variable number tandem repeat-Alus (SINE-VNTR-Alus or SVAs), and U6s^10^. While most TEs move throughout the genome without adverse consequences, they are occasionally inserted into critical regions, leading to disruption of transcription, splicing, or translation^8,10^.

Pathogenic MEIs have been demonstrated to cause a number of genetic conditions, including HBOC and other cancer predisposition syndromes^11^. The prevalence of pathogenic MEIs among disease-causing variants is estimated to be between 0.16 and 0.3%^8^; however, this is likely underestimated due to challenges with detection. Historic PCR amplification of sequencing targets might underrepresent or miss alleles containing these insertions due to size (under-amplification) or allele dropout. With improved coverage, capture-based next-generation sequencing allows for the detection of large copy number variants and rearrangements, such as TEs. However, detection requires data analysis optimization and functional confirmation^12^.

Here we report a case involving the identification of a previously undetected pathogenic MEI in *BRCA2* using an updated variant calling method in a patient with early-onset breast cancer.

A 40-year-old Emirati female presented with unilateral breast cancer. The initial biopsy revealed invasive ductal carcinoma (IDC), and it was estrogen (ER) and progesterone receptor (PR) positive and human epidermal growth factor receptor 2 (HER2) negative. Although there was no reported family history of HBOC-associated cancers (Fig. 1), she met National Comprehensive Cancer Network (NCCN) guidelines for *BRCA1*/*2* testing^2^. Expedited testing for *BRCA1*, *BRCA2*, and *PALB2* was ordered to inform surgical decisions. She expressed a preference for bilateral mastectomy should a PV be detected.

**Fig. 1:**
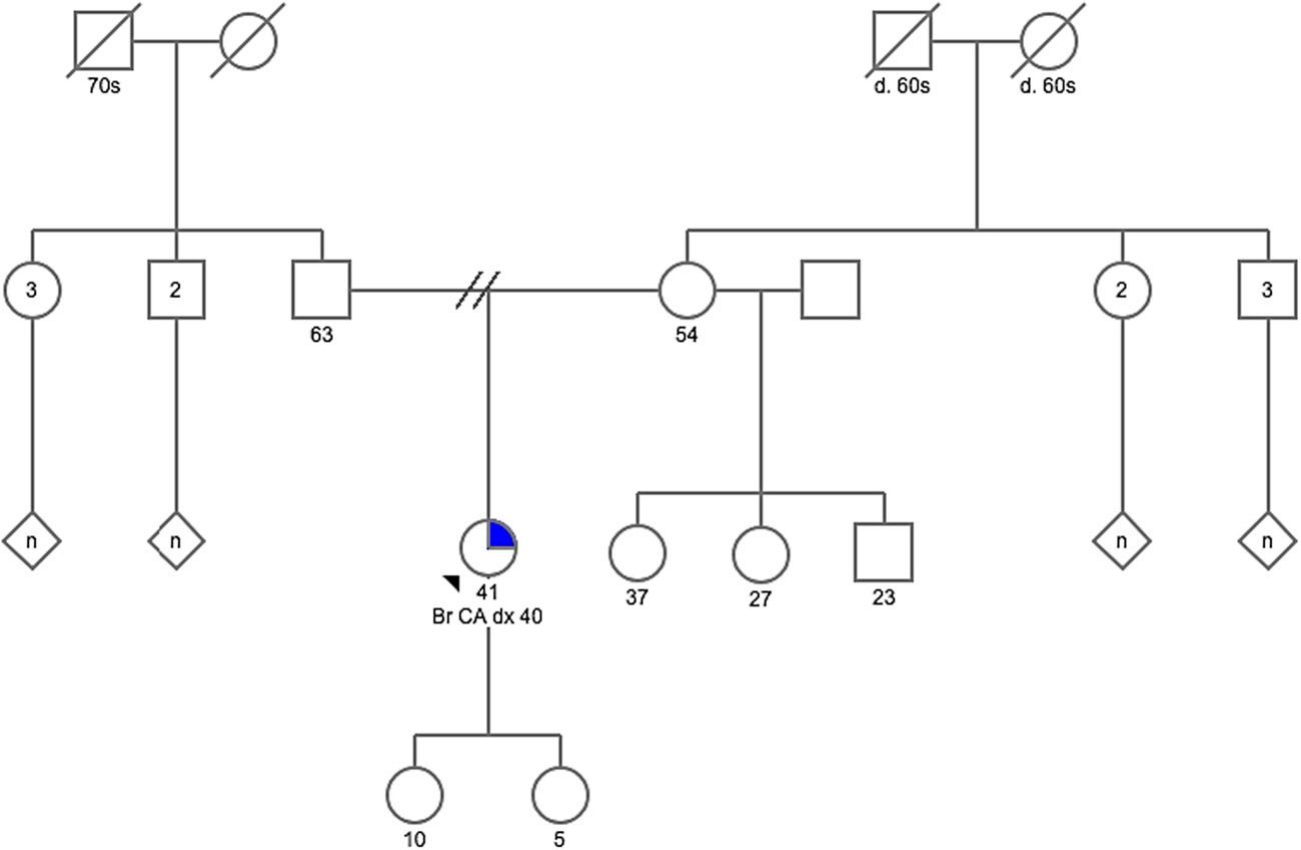
The patient’s pedigree reveals no known family history of hereditary breast, ovarian, or other related cancers. The proband is the only child to her parents; however, she has three half-siblings through her mother and numerous aunts, uncles, and cousins on both sides of the family. While this suggests that the MEI is de novo in the proband, it is possible that some members of the family may have the variant and have simply not developed cancer due to age-related penetrance. Her grandparents are reported to have died of noncancer-related causes in their 60s and 70s, and it is possible that they died before cancer would have developed. Only sequencing of family members could confirm the de novo status of the *BRCA2* MEI; however, samples were not available.

Testing revealed two variants that were classified by the testing laboratory as being of uncertain significance (VUS): *BRCA2* (NM_000059.3) c.6842G>A (p.Gly2281Glu) and *PALB2* (NM_024675.3) c.3464C>G (p.Ser1155Cys). Using ACMG variant classification guidelines, both variants had moderate evidence for pathogenicity (PM2) given their low frequency in population databases, such as GnomAD. In silico predictions provided benign supporting (BP4) evidence for the *BRCA2* and *PALB2* variants^13^. Thus current evidence is insufficient to determine the role of these two variants in disease. The patient consequently proceeded with unilateral mastectomy for the affected breast. The final histology was stage IIIA (T3 N1 M0) grade 3 IDC ER+/PR+/HER2−. Cascade testing was unavailable for her relatives, and first-degree relatives were considered to be at moderate risk for breast cancer given her diagnosis.

Eight months later, her sequence was reanalyzed using an updated bioinformatic pipeline with increased sensitivity for TE detection. This pipeline development, named Mobster, was built for MEI detection and run with default parameters with post hoc filtering requiring at least 15 supporting reads^14^. An ~2000 base pair SVA insertion (c.7666_7667insSVA) was detected in *BRCA2*, which is a retrotransposon composite element with a portion of SINEs, VNTRs, and backward Alu repeats. This SVA appears to be 5′ truncated and is missing the CCCTCT repeats. The presence of a 17-bp target site duplication, L1 endonuclease recognition sequence, and poly-A tail together indicate that this SVA insertion occurred by L1-mediated target-primed reverse transcription (Fig. 2). This novel variant appears to disrupt the *BRCA2* DNA-binding domain^15^ and is classified as likely pathogenic^13^. The variant was confirmed by Sanger sequencing.

**Fig. 2:**
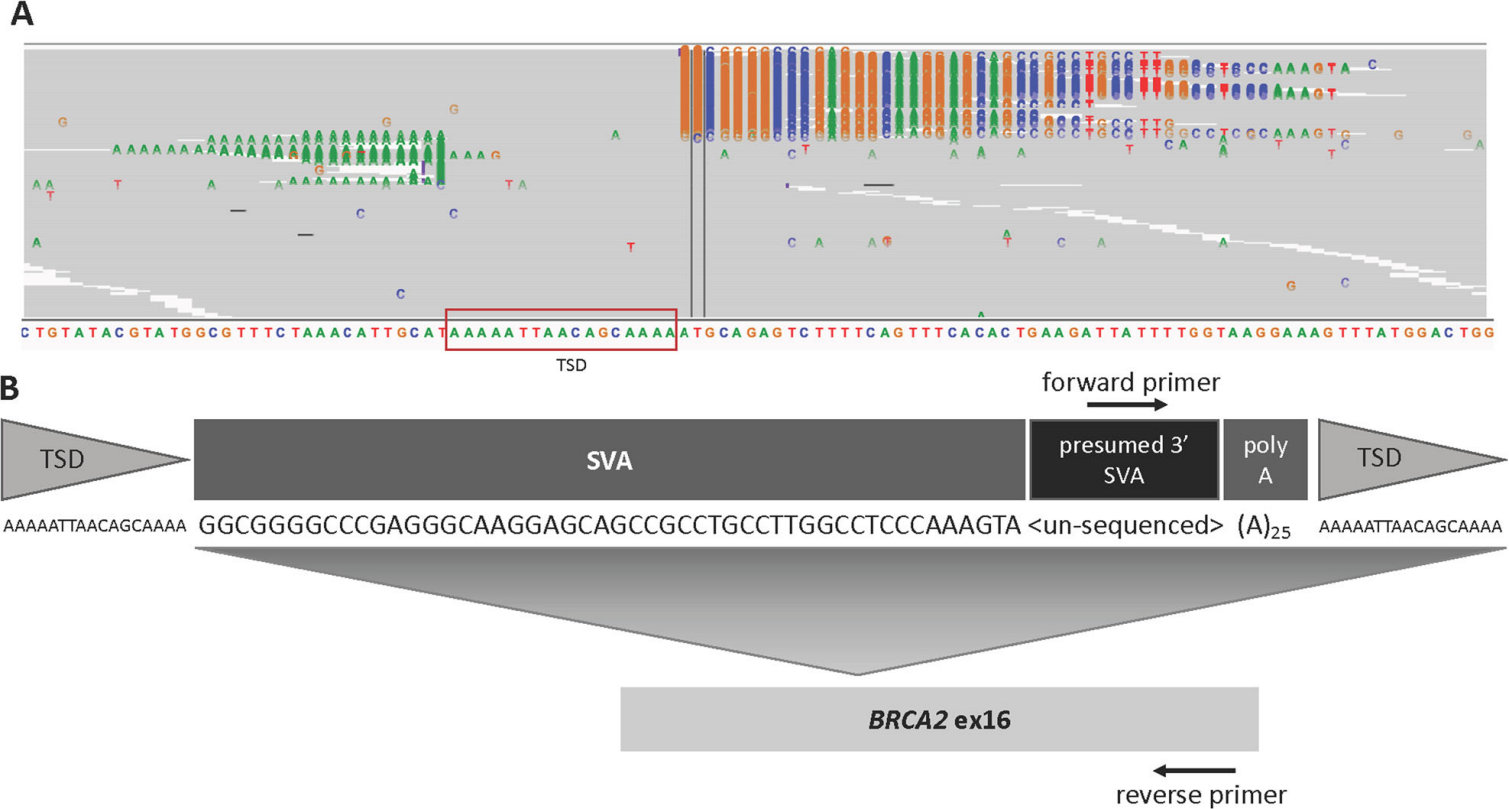
Molecular detection of the pathogenic MEI. **a** An IGV image from the proband. Colored bases indicate a mismatch relative to the reference genome. Strings of colored bases in a row indicate clipped reads at the SVA insertion breakpoint. The sequence on the bottom represents the reference genome. **b** Clipped reads at the insertion breakpoint were used to infer the structure of the SVA. A consensus sequence was built from both the right- and left-clipped read clusters. The right-clipped consensus represents the 5′ sequence of the SVA and demonstrates some 5′ truncation relative to an SVA-E sequence. The left-clipped consensus represents the poly-A tail of the sequence. The box labeled “presumed 3′ SVA” is not sequenced and not drawn to scale. If there are no additional rearrangements within the SVA sequence, then the nonsequenced portion is likely ~2kb long. A primer designed based on the SINE-R region of known SVA-E elements was used to amplify the 3′ insertion breakpoint.

This variant confirmed a diagnosis of HBOC, which had significant implications for risk management. Assuming that this variant conferred a similar risk to other *BRCA2* PVs, the patient’s contralateral breast cancer risk was approximately 35 and 53% at 10 and 20 years, respectively^1^. The patient was recommended to undergo annual mammograms and magnetic resonance imaging for her remaining right breast and may consider RRCM. Of significance, she was now considered to be at increased ovarian cancer risk (~17%; 95% confidence interval)^1^, and RRBSO was recommended by age 45 years^2^. She subsequently underwent total hysterectomy (for endometriosis) and RRBSO and opted for annual breast surveillance. Cascade testing was recommended for her at-risk relatives, although it was possible that the MEI was de novo in view of absent cancer family history (Fig. 1). Her children have a 50% chance of inheriting the variant and should consider testing as adults.

This case highlights the causative role MEIs can play in hereditary cancer syndromes. The overall prevalence of MEIs is likely underestimated and may be more common in some genes than in others^11^. One large cohort study found that RE insertions accounted for a larger proportion of PVs in *BRCA2* than in *BRCA1* and *APC*^11^. They postulated that this may be attributable to the larger size of *BRCA2*, as well as being a possible “*hot spot*” for oncogenic insertions^11^. The specific detected TE in this case (SVA) is larger and rarer than other Alu elements and is thought to comprise ~0.2% of the genome^11^. It is important to note, however, that SVAs also make up a disproportionate number of new MEIs given that this is an active family with active source elements. These SVAs have been reported in a number of other genes, but to date, only insertions in a few other genes, such as *PMS2* and *CASP8*, have been associated with increased cancer risk. This is the first report of an SVA in *BRCA2*. Optimized analysis specifically for TEs will improve our understanding of the prevalence of these variants and help us better assess which genes should be more stringently evaluated for TEs in clinical testing.

It is also important to test for MEIs in all patients with a clinical suspicion of hereditary cancer syndromes, regardless of family history. Previous studies of TEs in human disease have focused on family history and founder variants^11,16^. MEIs may present as de novo variants and should be considered if PVs are not initially detected by standard sequencing and deletion/duplication analysis. It is important to inquire whether the laboratory performing testing has the capability to detect MEIs. Thorough evaluation for MEIs in regions exhibiting evidence of insertion should be considered a routine part of clinical testing.

This is the first report of an MEI in a person of Arabic descent. There appears to be some correlation between ancestry and the likelihood of detecting a germline TE. The percentage of TE-based PVs may vary by ancestry, as Africans had a much higher proportion of TEs when compared to Europeans^11,16^. Founder variants have been previously described in a number of populations, including African and Latin/Caribbean populations, and most notably, the Portuguese *BRCA2* c.156_157insAlu variant^17^.

This case demonstrates the contributory role of MEIs in cancer predisposition syndromes and contributes to the body of literature implicating them as a mechanism for disease. Improvements in bioinformatic analysis with increased sensitivity for MEI detection are important for clinical decision-making. While MEIs may be more prevalent in certain genes or ethnicities^5,11^, they should be considered in all individuals with suspected cancer syndromes. Patients should be counseled that reanalysis of genetic sequencing may impact their results and future clinical management. There is a need for better guidelines for patients, clinicians, and laboratories on sequencing reanalysis and follow-up for VUSs and uninformative results^18^.

## Data Availability

The relevant data from this Data Report are hosted at the Human Genome Variation Database at 10.6084/m9.figshare.hgv.2888.
